# Rescue *In Vitro* Maturation in Polycystic Ovarian Syndrome Patients Undergoing *In Vitro* Fertilization Treatment who Overrespond or Underrespond to Ovarian Stimulation: Is It A Viable Option? A Case Series Study

**DOI:** 10.22074/ijfs.2020.6025

**Published:** 2020-07-15

**Authors:** Muhammad Fatum, Marie-Eve Bergeron, Caroline Ross, Anni Ding, Ayesha Bhevan, Karen Turner, Tim Child

**Affiliations:** 1 Oxford Fertility Unit, Institute of Reproductive Sciences, Oxford, United Kingdom; 2 Department of Obstetrics and Gynaecology, Faculty of Medicine, Centre Hospitalier Universitaire de Quebéc, Université Laval, Quebéc, QC, Canada

**Keywords:** Infertility, *In Vitro* Fertilization, *In Vitro* Maturation Techniques, Oocytes

## Abstract

**Background:**

This study intends to present the role of rescue *in vitro* maturation (IVM) in polycystic ovarian syn- drome (PCOS) patients undergoing *in vitro* fertilization (IVF) treatment who have inappropriate responses to ovarian stimulation.

**Materials and Methods:**

This was a retrospective case series study of five PCOS patients undergoing IVF treatment considered for cycle cancellation due to increased risk of ovarian hyperstimulation syndrome (OHSS) as group A or poor response to ovarian stimulation as group B. Patients in group A had high oestradiol levels and recruitment of high numbers of small/intermediate sized follicles that did not meet the criteria for human chorionic gonadotropin (hCG) triggering. Patients in group B responded inadequately to hormonal stimulation despite high gonadotropin dosage. Treatment was changed to rescue IVM cycles after the patients provided consent.

**Results:**

In group A, three IVF patients deemed to have high chances of developing OHSS as evidenced by high oestradiol levels were converted to IVM. A total of the 58/68 oocytes retrieved were mature or matured *in vitro*. There were 26 cleaving embryos obtained. Two patients had live births and one patient suffered a miscarriage. In group B, rescue IVM was implemented in two patients due to poor ovarian response (POR). A total of 22/26 oocytes retrieved were mature or matured *in vitro*. There were 13 cleaving embryos obtained. One patient had a live birth, whilst the other suffered a miscarriage.

**Conclusion:**

Rescue IVM could be a viable option in PCOS patients undergoing IVF treatment who are unable to safely meet the criteria for hCG triggering due to overresponse to ovarian stimulation or ovarian resistance to high doses of stimulation. Conversion to IVM can still result in reasonable oocyte retrieval and lead to clinical pregnancy and live births without the risks of OHSS.

## Introduction

Ovarian superovulation with gonadotropin stimulation is still the mainstay of *in vitro* fertilization (IVF) (1). The aim of ovarian stimulation is to induce multifollicular recruitment with as much synchronized cytoplasmic and nuclear maturation as possible, and to safely obtain a higher number of mature eggs at the time of egg collection (2). Side effects of ovarian stimulation can include breast tenderness, abdominal bloating, nausea and vomiting (3). More importantly, it can lead to ovarian hyperstimulation syndrome (OHSS), particularly in women with polycystic ovarian syndrome (PCOS) (4, 5).

PCOS is probably the most frequently encountered endocrinopathy in women of reproductive age (6). It is characterized by irregular menses, hyperandrogenism, and polycystic ovaries (PCO) on ultrasound findings. The prevalence of PCOS may be as high as 15-20% (7). It is believed that harvesting more eggs would compensate for subfertility in these patients. However, ovarian responses to the same stimulation protocols may vary considerably among different PCOS patients and even among different cycles in the same patient (8).

In some cycles, patients may be overstimulated, resulting in a very high number of growing follicles and increased levels of oestradiol. This group of patients is at higher risk of developing OHSS (9-11). In addition, a large cohort of antral and preantral follicles are recruited in these overstimulated cycles, which are asynchronous and heterogeneous in their growth and development (1). Consequently, immature and mature eggs are retrieved in these cycles. In some cases, this may prove to be a complex conundrum that needs much consideration, particularly when the patient is at high risk of OHSS, as demonstrated by high hormone levels, and there is an insufficient number of large-sized follicles. In these cases, cancellation could be the only option. Coasting may not be effective or plausible, as oestradiol production may increase further (12).

On the other end of the spectrum, management of PCOS women with poor ovarian response (POR) can be an equally frustrating challenge. Despite the high number of small follicles per ovary (2-3 times that of normal) (13), there is poor follicular growth and development in response to gonadotropin stimulation. This adversely affects mature oocyte retrieval and, more importantly, pregnancy success. Like patients at high risk of developing OHSS, these women also face the prospect of cycle cancellation.

We report a cohort of overstimulated IVF patients, as indicated by their rapidly increasing oestradiol levels and the large number of follicles, and a cohort of poor responders to ovarian stimulation who converted to rescue *in vitro* maturation (IVM) treatment. The aim of this study is to examine the rate of immature oocyte recovery and their potential for IVM from cancelled IVF cycles due to an abnormal response to gonadotropin stimulation.

## Materials and Methods

### Eligible patients

Unplanned IVM rescue cycles were undertaken for five PCOS patients who had abnormal responses to gonadotropin stimulation as part of their IVF treatment between 2007 and 2010 at the Oxford Fertility Clinic.

PCOS was defined according to the modified Rotterdam criteria (14) . Women who were considered to have overresponded had either high levels of oestradiol and/ or a high number of growing follicles (>20 at an early stage). Conversely, women who were considered as resistant to gonadotropin stimulation either responded poorly biochemically with low oestradiol levels or had poor follicular growth as evidenced by scans. Women aged over 40 and who had more than three previous failed IVF cycles were excluded from the study. In accordance with Oxford University Ethics Committee, the study was not registered and Ethical approval was not required as data were anonymised, not identifiable by researchers and were collected before the study was formulated.

### *In vitro* fertilization and *in vitro* maturation

Our standard protocol for IVF and IVM treatments were described previously (15).

### Statistical analysis

This was a case series study produced as part of an IVM programme at Oxford Fertility Unit, UK. Statistical analysis was carried out by a biostatistician at Oxford University. Statistical analyses were done using Microsoft Excel (Microsoft Office 365). Table was produced using Microsoft Excel (Microsoft Office 365). Graphs were produced using GraphPad Prism 8.0.0 on Mac OSX (Apple Inc. USA). The case series was reported using the case report (CASE) guidelines checklist (16).

## Results

We present five cases of PCOS patients (see criteria above) aged between 31 and 39 years who each underwent an unplanned rescue IVM cycle due to an abnormal ovarian response to gonadotropin stimulation at Oxford Fertility Clinic between 2007 and 2010. They agreed to undergo immature oocyte maturation retrieval with subsequent IVM of oocytes to rescue their IVF treatment. Prior to the treatment, they all had normal ovarian reserves according to their early follicular phase follicle stimulating hormone (FSH) and antral follicle counts (AFC). The main results examined were biochemical pregnancy [beta human chorionic gonadotropin (βhCG) positive], clinical pregnancy rate (defined as heart activity at 8 weeks on an ultrasonography scan) and live birth rate.

**Fig 1:**
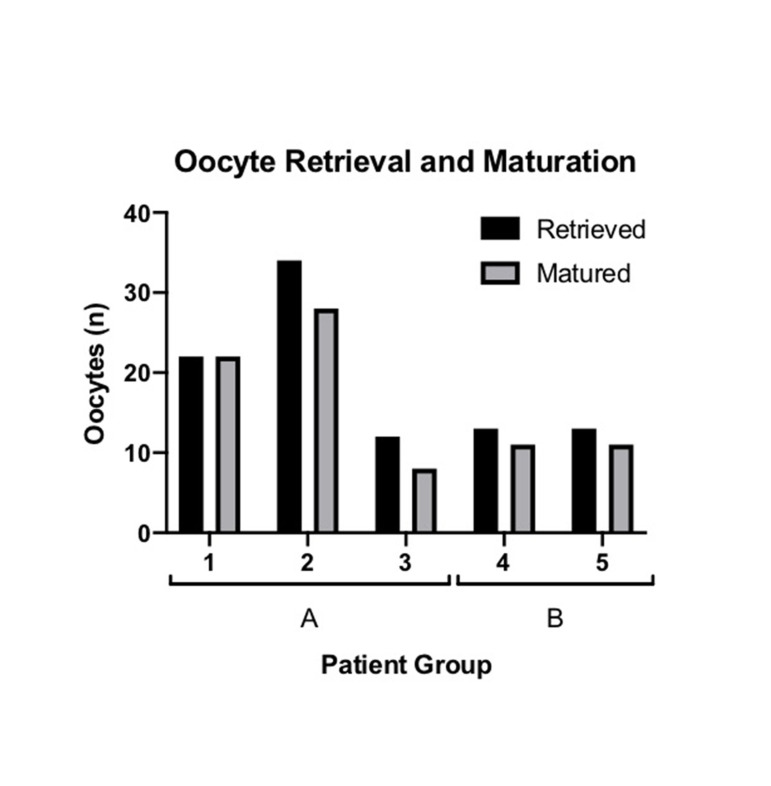
Numbers of oocyte retrieved and matured. Bar chart shows the numbers of oocytes retrieved and matured for each patient. Patients 1-3 represent group A and patients 4-5 represent group B.

Three patients (group A) were offered the option of converting to IVM rather than cancelling their IVF cycles as they were deemed to be at risk of developing severe OHSS. Average oestradiol on the day of cancellation was 11 078 ± 5141.9 pmol/L (Table 1). Nevertheless, none of these patients actually developed OHSS. Oocyte retrieval rate per aspirated follicle was 35%. A total of 68 oocytes were retrieved between the three patients in each group, and 58 of the 68 oocytes reached metaphase I (MI) or metaphase II (MII, Fig .1). Twenty-six cleaving embryos were obtained in group A (Table 1).

**Table 1:** ZKPQ score comparison between the CBRC and local groups according to IVF technique


Ptno.	Age	BMI	E2 onday ofcancellation	Oocytesretrieved	Oocytesreaching MIor MII(% of total)	No.ooc ytesinjected	Fertilizationrate	No.cleavingembryos	Embryostransferred	Pregnancytest	Cycleoutcome

1	32	23	6065	22	22 (100)	22	17 (77)	12	2	+	Live birth
2	31	21	16340	34	28 (82)	28	15 (54)	12	2	+	Miscarriage21 weeks
3	34	23	10830	12	8 (67)	8	4 (50)	4	2	+	Live birth
4	32	23	1800	13	11 (85)	11	6 (55)	5	2	+	Live birth
5	39	24	2483	13	11 (85)	11	8 (73)	8	2	+	Biochemicalpregnancy


Table showing baseline characteristics of each patient, oestradiol levels on the day of cancellation of IVF treatment, as well as parameters on oocytes and embryos obtained in each case. Patients 1-3 represent group A. Patients 3-4 represent group B. Pt; Patient, no; Number, MI; Metaphase I, MII; Metaphase II, BMI; Body mass index, and IVF; *In vitro* fertilization.

In group B, two patients were offered the option of rescue IVM cycle because they had POR to gonadotropin stimulation. Average oestradiol level of the day of cycle cancelation was 2141.5 ± 482.9 pmol/L (Table 1). Despite their disappointing response to ovarian stimulation, 13 oocytes were retrieved from each patient. In fact, oocytes could be obtained in 33% of all follicles identified and aspirated. Eleven oocytes were mature or matured *in vitro* for each patient (Table 1). A total of 13 cleaving embryos were obtained in this group.

In both groups, all patients had two fresh cleavage embryos transferred on day 3 of development and all (100%) had positive pregnancy tests two weeks later. Three of the five patients (60%) gave birth to healthy singletons at term (38 and 40 weeks) or near term (35 weeks). Unfortunately, one patient in group A had a late second trimester miscarriage and one patient in group B had an early first trimester miscarriage (Table 1). Moreover, three patients had the opportunity to store their embryos. Two patients returned for a total of three frozen embryo replacement cycles, but they were all unsuccessful.

## Discussion

Our case series study shows that rescue IVM could be a viable option in PCOS patients undergoing IVF treatment but failing to safely meet the criteria for hCG triggering because of either ovarian overresponse or underresponse to hormonal stimulation.

In our study, we did not use the conventional definition of POR as defined by the European Society of Reproduction and Embryology (ESHRE) (17). Instead, POR in our study referred specifically to PCOS patients with normal ovarian reserve and high AFC, yet showed poor hormonal and follicular response despite controlled ovarian hyperstimulation (COH). POR patients have reduced oocyte production, cycle cancellation and, most importantly, a reduced probability of pregnancy. It is unclear why women with PCOS can have such contrasting responses to gonadotropin stimulation, although it has been suggested that certain PCOS phenotypes may be correlated with adverse assisted reproductive outcomes (8). There is no test that can reliably predict outcome of ovarian stimulation in women with PCOS. However, anti-Müllerian hormone (AMH) on day 3 of the IVF stimulation cycle may positively predict ovarian response to gonadotropin stimulation. Oestradiol levels on the day of hCG administration and oocyte retrieval rate positively correlate with increasing AMH levels during IVF cycles in PCOS patients (18). As there is no way to reliably predict poor responders to gonadotropin stimulation, we cannot immediately identify these women for IVM. However, rescue IVM after failed IVF may provide these women with a chance of pregnancy within the same cycle of treatment.

There have been efforts to identify an algorithm based on the woman’s age and markers of ovarian reserve to optimise the FSH starting dose in assisted reproductive techniques (ARTs). A recent study suggested that the application of a nomogram could lead to a more tailored approach, increasing the cost-effectiveness of infertility treatment. In general, the starting dose of FSH as calculated by the nomogram was lower than the actual prescribed dose, which might reduce the risk of OHSS. However, the authors also suggested the inadequacy of the nomogram in PCOS patients, especially in those with high AMH levels (19). Further studies are required to assess the utility and generalisability of such nomograms. The risk of OHSS may also be reduced by the administration of adjuvant medication. Administration of D-chiro-inositol (DCI) in PCOS patients resulted in a higher ovulation rate compared to placebo (20, 21). Myo-inositol and DCI may improve many of the metabolic and hormonal dysregulations characteristic of PCOS (22), and myo-inositol seems to be able to increase oocyte quality, decrease the days of FSH stimulation before hCG administration and, hence, the risk for OHSS (23, 24).

OHSS is an iatrogenic, systemic condition secondary to gonadotropin stimulation that occurs either during the luteal phase or during pregnancy. The most common form happens a few days after the induction of follicular rupture via injection of hCG when follicular growth has been medically induced (25). Fundamentally, in OHSS, an increase in vascular permeability results in third-space fluid loss, leading to intravascular volume depletion and haemoconcentration (9). Thromboembolism is a potentially serious consequence of OHSS, and can sometimes be fatal despite treatment (26). Additionally, OHSS been reported to be linked to hepatic and renal dysfunction (27, 28), but the link between COH and renal/ liver dysfunction are still debated. A study by Romito et al. (29) examined 426 patients undergoing IVF treatment and found that COH did not significantly alter renal and hepatic functions. In contrast, Giugliano et al. (30) reported a case of hepatic failure after four cycles of COH in a patient that developed severe haemolysis, elevated liver enzymes and low platelets (HELLP) syndrome. Various preventative strategies of OHSS during IVF have been suggested, such as coasting (31), co-treatment with cabergoline (32) or metformin (33), cryopreservation of embryos (34), or the administration of gonadotropin releasing hormone agonists (GnRH-agonist) instead of hCG in women treated in antagonist protocols (35). However, the only absolute way of preventing OHSS is to avoid ovarian stimulation, as in IVM. Given the evidence between COH and renal/ liver dysfunction is still debated, avoiding ovarian stimulation by using IVM may have the added advantage of preventing such complications, especially when many women have already gone through multiple cycles of IVF and may be at higher inherent risk for developing renal/ hepatic dysfunction.

Despite the advances in ARTs, one of the main challenges is the management of patients who have POR. To this end, luteal phase ovarian stimulation and dehydroepiandrosterone (DHEA) supplementation have shown promising results in improving outcomes in PORs. Preliminary results from a single centre pilot study by Lin et al. have demonstrated that luteal phase ovarian stimulation significantly improved oocyte retrieval and quality when compared to follicular phase ovarian stimulation in patients undergoing IVF (36). In a similar finding, Chern et al. (37), in their retrospective study, reported a potential benefit of DHEA supplementation pre-IVF cycle in PORs by showing improved oocyte retrieval rate, quality of embryos and live birth rate compared to the control group.

The success rate with IVM is associated with the number of immature oocytes obtained, which is predicted by the AFC. Women with PCOs have higher AFCs (13) and, therefore, have a comparatively increased rate of success than those with normal ovaries. Women with PCO are at significantly higher risk of developing OHSS (4, 5). In our previous study, we have reported that IVM is a simpler, safer, although less successful alternative, for women with PCO or PCOS (15). Balancing the higher success rate of IVF in PCO/PCOS women with the risk of potentially developing OHSS can be a complex dilemma. With the possibility of initial IVF treatment, and then rescue IVM if they are at significant risk of developing OHSS, we may be able to make a compromise between success rate and safety that neither IVF nor IVM alone can achieve in PCOS patients. One of the strengths of our study is the corroboration of previous findings, not only from our own group but that of others. The concept of rescue IVM began approximately two decades ago. Coskun et al. (38) have demonstrated that immature oocytes can be recovered from cancelled human gonadotropin cycles and these oocytes can be matured *in vitro*. Later, in a related publication, Jaroudi et al. (39) reported on 18 patients who underwent IVF but were then deemed to be at significant risk of developing OHSS. These women had cycle cancellation and underwent immature oocyte retrieval with subsequent IVM. On average, 8.1 immature oocytes were retrieved from each patient and 44 embryos were transferred in 17 cycles. There were two live births; however, one baby was delivered preterm and died shortly after. The study suggested that oocytes matured *in vitro* from incomplete IVF cycles could be fertilised by intracytoplasmic sperm injection (ICSI) and the those embryos could result in pregnancies. However, at the time, the low success rate could not justify recommendation of more widespread use without further research. In our study, the average number of oocytes retrieved per patient in both groups was higher than reported by Jaroudi et al. (39).

There are a number of potential explanations for this. First, the study by Jaroudi et al. (39) included not only PCOS patients, but also those with other types of infertility, such as anovulatory and unexplained cases. It is known that PCOS patients have higher numbers of follicles from which immature oocytes may be retrieved. It is also plausible that the improvements in both the IVF and IVM protocols have contributed to the higher numbers of immature oocytes picked up in our study. The live birth rate (60% overall) in our study was also higher. Again, improvements in techniques and protocols may have contributed to results; however, we are aware that our cohort is very small. In our study, the maturation rate (reaching MII) in group B (27%) was lower than that in group A (58%), which was comparable with our previous study (65%) (40). Whilst this seems to be a significant difference, it is noteworthy that the cohort size in our previous study was 94, which is considerably larger than that of our current study. It is possible that there a genuine difference exists in the ability of oocytes to mature between poor responders and overresponders, which may share the same aetiology as ovarian resistance to hormonal stimulation. The fertilization rate for both groups is similar to that reported in our previous study, which is promising as it suggests that oocytes in rescue IVM are not adversely affected by their previous exposure to gonadotropin sti mulation, regardless of the ovarian response.

The main limitation of our study is the sample size the high clinical pregnancy rate and live birth rate requires caution. Whilst a biostatistician carried out the data analysis, we did not calculate the sample size required before the start of the study. This was due to logistical reasons of finding cases of cancelled IVF with subsequent agreement of undergoing IVM. Arguably this affects the generalisability of our study and the ability to draw definitive conclusions based on the findings of this mini case series. However, our aim is to highlight the possibility of IVM success in a proportion of PCOS patients who fail IVF treatment in a field that has the scope for further study and research.

IVM has an inherent advantage over conventional IVF by utilising the natural menstrual cycle, and bypassing the need for ovarian stimulation and pituitary suppression, albeit at the cost for reduced chances of success. Conventionally, IVM has been considered an alternative to IVF in women at risk of OHSS or in those who may have a POR to gonadotropin stimulation. Here, we present IVM as a potential add-on treatment, which is not considered as an alternative to IVF, but rather alongside it as a rescue strategy. The advantage is that potentially recoverable immature oocytes in cancelled cycles are not wasted and the emotional stress associated with facing a potentially cancelled cycle is reduced. Additionally, it may help prevent these patients from undergoing another costly, lengthy stimulation protocol.

## Conclusion

We conclude that rescue IVM could be a viable option in PCOS patients undergoing IVF treatments who fail to safely meet the criteria for hCG triggering, either due to overresponse to ovarian stimulation or ovarian resistance to high doses of stimulation. Conversion to IVM can still result in reasonable oocyte retrieval and lead to clinical pregnancy and live births without the risks of OHSS. Further research is needed to determine the aetiology of POR and OHSS, and identify markers that will allow us to reliably predict which patients for whom IVF is less appropriate than IVM. Larger studies are needed to determine whether rescue IVM is a widely applicable strategy for women who respond inappropriately to ovarian stimulation and its success rate.
